# An interpretable machine learning model integrating 54 test indicators and age: a single-center study on precise discrimination between serous ovarian cancer and ovarian cysts

**DOI:** 10.1186/s12911-026-03727-3

**Published:** 2026-08-08

**Authors:** Chunmei Liu, Zhongning Wei, Yang Qiu, Ying Ju, Fulu Chu, Bingru Lu, Shan Ding, Yiqing Liu

**Affiliations:** 1 Department of Clinical Laboratory, Shandong Provincial Hospital Affiliated to Shandong First Medical University, 324 Jingwuweiqi Rd, Huaiyin, Jinan, Shandong 250021 China; 2 Department of Clinical Laboratory, Dongming County People’s Hospital, Heze, Shandong 274500 China

**Keywords:** Serous ovarian cancer, Ovarian cyst, Machine learning, Prediction model

## Abstract

**Background:**

Serous ovarian cancer (SOC) is a highly malignant subtype of epithelial ovarian tumors, most patients are diagnosed at an advanced stage (III/IV). However, existing single tumor markers have limited diagnostic performance. Therefore, this study aims to develop an interpretable machine learning model by integrating 54 test indicators and age to accurately differentiate between SOC and ovarian cysts.

**Methods:**

A retrospective analysis was conducted on 648 patients with pathologically confirmed diagnoses (SOC = 155 cases, benign cysts = 493 cases). The i-Research software developed by Roche, based on R language, was used to randomly divide the dataset into a training set and a validation set at a 7:3 ratio. An integrated feature selection approach combining LASSO, Random Forest (RF), and Decision Tree algorithms was applied to identify six key indicators for model construction. Eleven machine learning algorithms, including logistic regression (LR), RF, and XGBoost, were evaluated using eight performance metrics to identify the optimal model. SHAP values were employed to provide multi-level interpretability.

**Results:**

The LR model was selected as the final model because of its better interpretability and clinical usability on the internal validation set (*n* = 195): AUC = 0.979 (95% CI: 0.958–0.994), sensitivity = 89.4%, specificity = 93.9%, accuracy = 92.8%, F1 score = 92.9%, and Kappa coefficient = 80.9%. Age, RAR, CA125, CA153, and MLR were identified as risk factors, whereas RBC count was found to be a protective factor.

**Conclusion:**

This study innovatively constructs an interpretable LR model offering a practical and reliable tool for the preoperative differential diagnosis of SOC and ovarian cysts.

**Clinical trial number:**

Not applicable.

**Supplementary Information:**

The online version contains supplementary material available at https://doi.org/10.1186/s12911-026-03727-3.

## Background

### Research background

Ovarian cancer is the third most common gynecological malignancy but the leading cause of cancer-related death in women [1]. Epithelial ovarian cancer (EOC), accounting for approximately 90% of all ovarian cancers, carries the highest case-fatality rate among reproductive tract malignancies [2]. Serous ovarian cancer (SOC), the predominant EOC subtype comprising ~ 75% of cases, is particularly aggressive with high metastatic potential and poor prognosis [3]. Because the ovaries lie deep within the pelvic cavity and early-stage SOC lacks specific symptoms and effective screening modalities, most patients are diagnosed at advanced stages (III/IV) with metastasis [4]. In contrast, ovarian cysts are common benign lesions, especially prevalent among women of reproductive age. Most ovarian cysts are physiological or benign and require only monitoring or minimal treatment. Accurate early differentiation between SOC and benign ovarian cysts is essential to prevent both unnecessary surgery and treatment delays, yet this remains a formidable clinical challenge.

Current differential diagnosis relies primarily on ultrasound examinations, serum tumor marker testing, and pathological analysis [5]. However, these methods are highly operator-dependent, and atypical cases remain prone to misdiagnosis or missed diagnosis [6]. Although Magnetic Resonance Imaging (MRI) and Computed Tomography (CT) provide superior soft tissue contrast and comprehensive assessments (e.g., peritoneal metastasis and lymph node status) [7], their high cost and limited accessibility restrict their use as first-line screening tools.

Carbohydrate antigen 125 (CA125) and human epididymis protein 4 (HE4) represent the most widely used serum biomarkers for ovarian cancer detection. While CA125 demonstrates high sensitivity for epithelial ovarian cancer, its specificity is compromised by elevations in various benign conditions, including endometriosis, pelvic inflammatory disease, and physiological states such as menstruation and pregnancy [8–10]. HE4, a more recent biomarker, offers improved specificity over CA125, although it may also be elevated in benign lesions such as endometriotic cysts. The integration of CA125 and HE4 with menopausal status into the Risk of Ovarian Malignancy Algorithm (ROMA) index has significantly improved diagnostic accuracy [11]. However, the ROMA index remains limited in certain complex cases, and most studies continue to rely on a narrow panel of markers, thereby underutilizing the rich multidimensional data generated in routine clinical practice.

Artificial intelligence (AI), particularly machine learning and deep learning, shows great potential in medical image analysis and multi-omics data integration. In ovarian cancer research, prediction models incorporating radiomics, genomics, or clinical data have shown promising results. Wu et al. developed a deep convolutional neural network for classifying ovarian tumor types in ultrasound images, achieving an overall accuracy of 95.2% [12]. While researchers at the University of Iowa demonstrated that machine learning-based analysis of DNA methylation patterns could accurately predict high-grade serous carcinoma (HGSC) using only nine methylation probes [13]. Despite these advances, most existing studies prioritize imaging or genomic data, largely overlooking the diagnostic potential of routine laboratory parameters—including inflammatory markers, coagulation profiles, liver and renal function tests, and sex hormone levels. Moreover, many complex models depend on specialized and difficult-to-obtain data (e.g., genomic data or specific imaging sequences), which limits their applicability in general clinical settings [14].

An ideal diagnostic model for ovarian cancer should simultaneously achieve high accuracy, clinical accessibility, interpretability, and ease of implementation. Existing approaches, however, are constrained by two major limitations: (1) an overreliance on a small set of classical tumor markers (e.g., CA125 and HE4), neglecting the potential contributions of numerous other routine clinical laboratory indicators; and (2) dependence on specialized data types that are not readily available in most clinical environments, thereby hindering widespread adoption [15].

### Current development status

Recently, diagnostic paradigm for ovarian cancer has gradually transitioned from reliance on single biomarkers to multi-dimensional integrated analysis. This evolution aims to overcome the limitations of traditional diagnostic methods and improve both diagnostic accuracy and early detection capabilities.

In the field of tumor marker research, an increasing number of biomarkers have demonstrated diagnostic potential for ovarian cancer. CA199 is frequently elevated in mucinous ovarian cancer [16]; alpha-fetoprotein (AFP) holds specific diagnostic value for ovarian yolk sac tumors [17]; human chorionic gonadotropin (β-hCG) serves as a critical reference marker for non-pregnancy-related choriocarcinoma [18]; and inhibin B contributes to the diagnosis of ovarian granulosa cell and theca cell tumors. Notably [19], these markers exhibit not only distinct expression profiles across different pathological types of ovarian tumors but also show quantitative differences between benign and malignant lesions. This provides a theoretical basis for enhancing diagnostic specificity through multi-marker combination analysis.

The application of machine learning in ovarian cancer diagnosis has progressed well beyond traditional logistic regression (LR), now spanning a wide spectrum of algorithms from random forest and support vector machines to sophisticated deep learning architectures. For instance, a decision tree (DT) model incorporating clinical indicators and preoperative blood cell parameters has demonstrated superior discriminatory performance in distinguishing benign from malignant ovarian tumors compared to single-modality imaging or biomarker assessments [20].

In another study analyzing a multimodal dataset from patients with advanced high-grade serous ovarian cancer, quantitative features—including nuclear size in hematoxylin and eosin-stained tissue and texture features from contrast-enhanced CT—were identified as prognostic indicators [21]. The integration of histopathology, radiology, and clinical genomics within machine learning models offers a promising strategy for enhancing cancer risk stratification through multi-modal data fusion. Collectively, these studies underscore the value of machine learning in ovarian cancer diagnosis and indicate that model performance can be further optimized through effective feature selection.

However, current research faces several limitations: small sample sizes and retrospective designs restrict generalizability; model validation lacks external validation; complex models require specialized data (e.g., methylation data or specific imaging sequences) unavailable in routine practice; and critically, model interpretability remains insufficient for clinical transparency [22].

To address these gaps, we developed a high-performance and interpretable machine learning model by integrating 54 routinely available clinical laboratory indicators and age to differentiate high-grade SOC from ovarian cysts. These readily accessible indicators -including tumor markers, blood counts, inflammatory indices, coagulation function, liver and kidney function tests, and sex hormone function tests- ensure the model’s clinical applicability and translational potential. The SHapley Additive exPlanations (SHAP) framework, a recent advancement in explaining tree-based models, is employed to provide both global and local interpretability, clarifying the contribution of each feature to individual predictions and enhancing clinicians’ understanding and trust in the model [23].

This study is expected to offer a practical decision-support tool tailored to this specific clinical scenario, thereby reducing unnecessary interventions and enhancing preoperative differential diagnostic rates.

## Methods

### Study design and patient cohort

This was a single-center, retrospective diagnostic cohort study. The protocol was conducted in strict accordance with the TRIPOD (Transparent Reporting of a multivariable prediction model for Individual Prognosis or Diagnosis) statement guidelines for reporting. This study was approved by the Ethics Committee of Shandong Provincial Hospital Affiliated to Shandong First Medical University (SWYX: NO.2023 − 419), and all participants provided written informed consent.

We consecutively enrolled patients treated at Shandong Provincial Hospital Affiliated to Shandong First Medical University who underwent surgical treatment between January 2016 and December 2022. All diagnoses were confirmed pathologically postoperatively.

Overall, 648 female patients were included, categorized as follows:

- Case group (SOC group): 155 patients with pathologically confirmed serous ovarian carcinoma (SOC).
- Control group (cyst group): 493 patients with pathologically confirmed benign ovarian cysts (including serous cystadenoma, mucinous cystadenoma, simple cysts).

Inclusion criteria: ① Completion of a full set of 54 preoperative laboratory tests at our hospital; ② Definitive pathological diagnosis following surgery; ③ Availability of complete clinical records. Exclusion criteria: ① Histopathological type did not match any of the abovementioned two types. (e.g., mucinous cancer, endometrioid cancer); ② Presence of other malignant tumors; ③ Coexisting severe hematological, immune system diseases, or active infections. The overall study workflow is summarized in supplemental Table 1.

To minimize selection bias, we employed consecutive patient enrollment from January 2016 to December 2022, including all eligible patients who met the criteria during this period. We did not use convenience sampling or exclude cases based on data completeness after initial screening. To prevent verification bias, all diagnoses were confirmed by pathological examination reviewed by two independent pathologists. Discrepancies were resolved by consensus. We acknowledge that, due to the retrospective nature and single-center setting, we were unable to conduct temporal validation to assess spectrum bias, which remains a limitation.

### Data collection and preprocessing

Data were extracted from the hospital’s electronic medical record system (EMR) and laboratory information system (LIS), including:

- Basic characteristics: age.
- Laboratory test indicators (total of 54):

- ◦ Tumor markers (5): CA125, CA724, CA153, CEA, AFP.
- ◦ Blood routine indicators (20): white blood cell count (WBC), red blood cell count (RBC), hemoglobin (HGB), platelet count (PLT), absolute neutrophil count (NEUT#), absolute lymphocyte count (LYMPH#), absolute monocyte count (MONO#), absolute eosinophil count (EO#), absolute basophil count (BASO#), and their respective percentages (NEUT%, LYMPH%, MONO%, EO%, BASO%), hematocrit (HCT), mean corpuscular volume (MCV), mean corpuscular hemoglobin (MCH), mean corpuscular hemoglobin concentration (MCHC), red blood cell distribution width coefficient of variation (RDWCV), red blood cell distribution width standard deviation (RDWSD).
- ◦ Inflammatory, immune, and derived indicators (9): Neutrophil-to-lymphocyte ratio (NLR), platelet-to-lymphocyte ratio (PLR), monocyte-to-lymphocyte ratio (MLR), systemic immune-inflammation index (SII = PLT × NEUT / LYMPH), inflammation index (neutrophil percentage-to-albumin ratio, NPAR = NEUT% × 100 / ALB), red blood cell distribution width standard deviation-to-albumin ratio (RAR = RDWSD / ALB), derived score index (hemoglobin-albumin-lymphocyte-platelet score, HALP = HGB × ALB × LYMPH / PLT), derived index (systemic inflammation response index, SIRI = MONO × NEUT / LYMPH), aggregate index of systemic inflammation (AISI = NEUT × PLT × MONO / LYMPH).
- ◦ Biochemical and liver function indicators (15 items): Total protein (TP), albumin (ALB), globulin (GLOB), albumin/globulin ratio (A/G), total bilirubin (TBIL), direct bilirubin (DBIL), indirect bilirubin (IBIL), alanine aminotransferase (ALT), aspartate aminotransferase (AST), gamma-glutamyl transferase (GGT), alkaline phosphatase (ALP), lactate dehydrogenase (LDH), glutamate dehydrogenase (GLDH), adenosine deaminase (ADA), total bile acid (TBA).
- ◦ Renal function indicators (5 items): Blood urea nitrogen (BUN), creatinine (CREA), blood urea nitrogen-to-creatinine ratio (BUN/CREA), uric acid (UA), cystatin C (Cys-C).

All the aforementioned test indicators were quantified with hospital-certified automated analyzers operating under stringent internal and external quality-control protocols. The established ovarian-cancer biomarker HE4 was excluded in this study due to the high rate of missing data (> 20%) in the institutional database. In addition, for the small proportion of missing values (< 10%), median imputation was applied to maintain dataset completeness. Additionally, 24 items exhibiting < 10% missingness were listed in supplemental Table 2. The remaining 29 variables exhibited no missing data (missing rate = 0).

### Feature engineering and variable selection

To identify the most predictive variables from the high-dimensional dataset and minimize the risk of overfitting, we implemented a systematic, multi-stage feature selection approach:

1. LASSO regression screening: We performed Least Absolute Shrinkage and Selection Operator (LASSO) regression to reduce dimensionality amid abundant baseline indicators. Relying on L1 regularization, LASSO mitigates multicollinearity in high-dimensional datasets and generates a sparse panel of candidate predictors. The optimal regularization parameter −logλ was selected via 5-fold cross-validation, and only variables with non-zero regression coefficients were retained for subsequent analysis.
2. Tree-based feature importance verification: RF and DT were subsequently applied to validate LASSO-selected features through tree-based importance rankings, which capture non-linear relationships and feature interactions that LASSO (a linear method) might miss.
3. Univariate and multivariate analysis: We conducted univariate and multivariate LR analyses (or Fisher’s exact test for categorical variables) to compare differences between the SOC group and the cyst group. This ensemble approach mitigates the limitations of any single method—LASSO alone might exclude features with non-linear effects, while RF alone might overfit to noise in high-dimensional spaces.

### Machine learning model development and validation

The entire cohort (*N* = 648) was randomly divided into a training set (*n* = 453) and an internal validation set (*n* = 195) at a 7:3 ratio. No significant differences in disease distribution (SOC vs. ovarian cysts) were observed between the two groups (*P* > 0.05).

Using the training set, we optimized hyperparameters through a 5-fold cross-validation grid search strategy and built prediction models for the following 11 machine learning algorithms:

- Naive Bayes (NB)
- K-Nearest Neighbors (KNN)
- Logistic Regression (LR)
- Random Forest (RF)
- Decision Tree (DT)
- Artificial Neural Network (ANN)
- Support Vector Machine (SVM)
- Gradient Boosting Decision Tree (GBDT)
- Light Gradient Boosting Machine (LightGBM)
- Adaptive Boosting (AdaBoost)
- Extreme Gradient Boosting (XGBoost)

Model performance was evaluated on the internal validation set using the following metrics:

Discriminative performance: Area Under the Receiver Operating Characteristic Curve (AUC), optimal threshold, accuracy, specificity (negative recall), sensitivity (positive recall), positive predictive value (positive precision), negative predictive value (negative precision), F1 Score (harmonic mean of precision and recall), and Kappa coefficient.

Calibration performance: Calibration curves were plotted and the Brier score was calculated to assess model calibration.

Clinical utility: Decision Curve Analysis (DCA) was performed to evaluate the clinical net benefit across different threshold probabilities.

### Model interpretation

To enhance the interpretability of the best-performing model and overcome the “black box” limitation inherent in machine learning, we employed the SHAP framework for global model interpretation. A SHAP summary plot was generated to illustrate the overall direction and magnitude of each feature’s influence on the model output.

### Statistical analysis

Continuous variables were summarized as median (interquartile range) [M(P25, P75)] and compared between groups using the Mann-Whitney U test. Categorical variables were expressed as frequency (percentage) [n (%)], and intergroup differences were analyzed using the chi-square test or Fisher’s exact test, as appropriate. All statistical tests were two-sided, and statistical significance was defined as *P* < 0.05. Statistical analyses were conducted using i-Research version 2.9.2 (developed by Zhou Weicheng of Roche) and SPSS 27.0 software.

## Results

### Variable selection and patient baseline characteristics

**Fig. 1:**
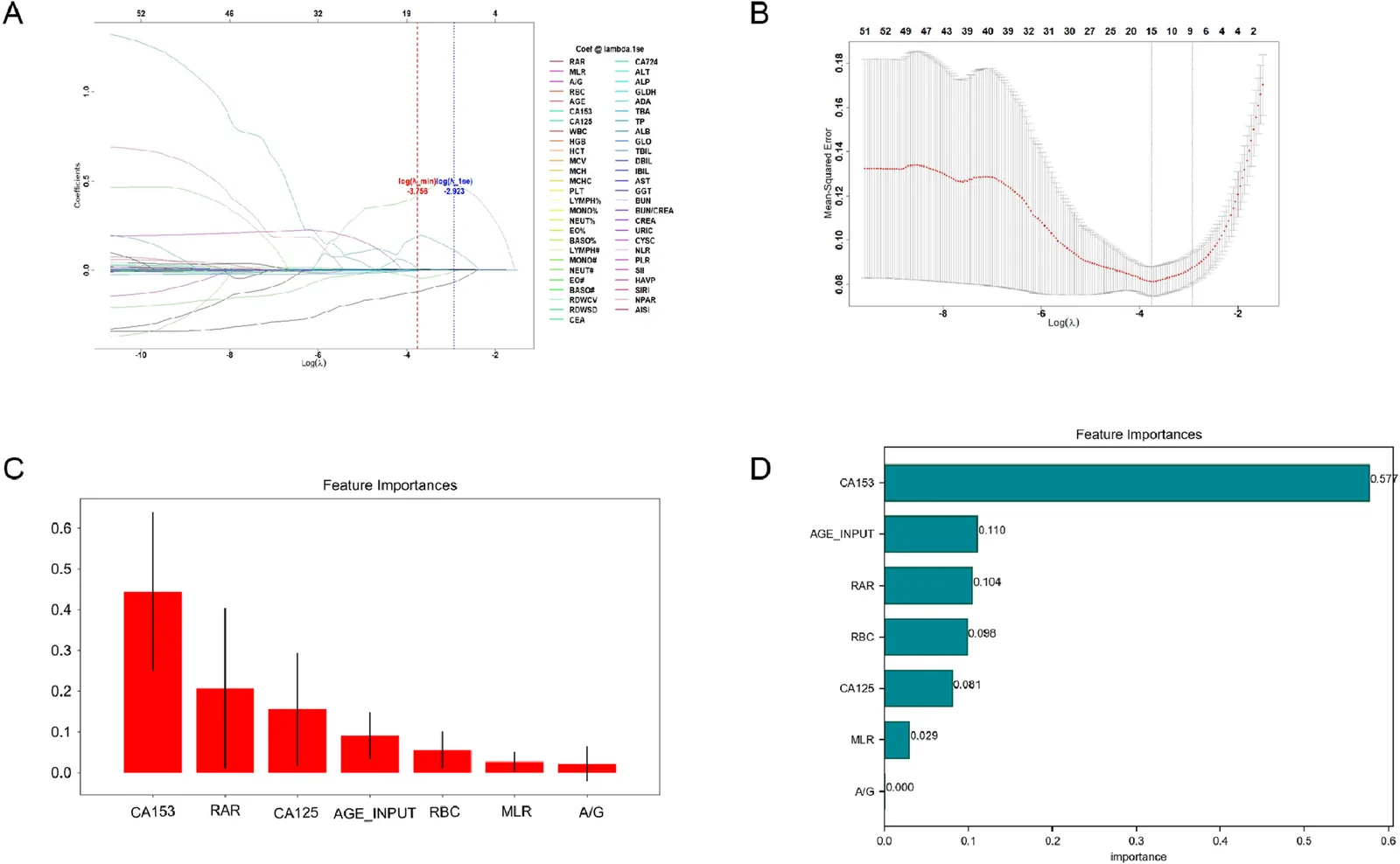
Indicator screening path diagram. (**A**) Model coefficient compression path plot. Each line corresponds to a variable, and the Y-axis denotes the regression coefficient of respective variable. As λ rises, the coefficients shrink progressively. The coefficients of several non-significant variables rapidly shrink to zero, indicating they will be excluded from the model. (**B**) Cross-validation curve for optimal model selection. The X-axis shows the −logλ of the penalty coefficient, and the Y-axis represents the likelihood deviation. Lower Y-axis values correspond to superior model fitting performance. (**C**) Feature importance derived from the RF algorithm. (**D**) Feature importance of the DT algorithm

LASSO regression initially selected seven key predictors from the original 54 indicators. As illustrated in Fig. 1, panel (A) shows the coefficient path diagram, where each line represents a variable’s regression coefficient along the y-axis. As λ increases, the coefficients of less important variables shrink toward zero, and are effectively excluded from the model. Panel (B) presents the cross-validation curve, with − log(λ) on the x-axis and the binomial deviance on the y-axis; lower deviance indicates superior model fit.

Furthermore, we employed RF and DT algorithms to assess the importance rankings of the abovementioned seven key predictors, and found that the A/G demonstrated the lowest contribution in both models and was accordingly excluded. This was shown in Fig. 1(C) and Fig. 1(D). All six remaining indicators showed statistically significant differences (*P* < 0.001), as presented in supplemental Tables 3 and supplemental Table 4.

**Fig. 2:**
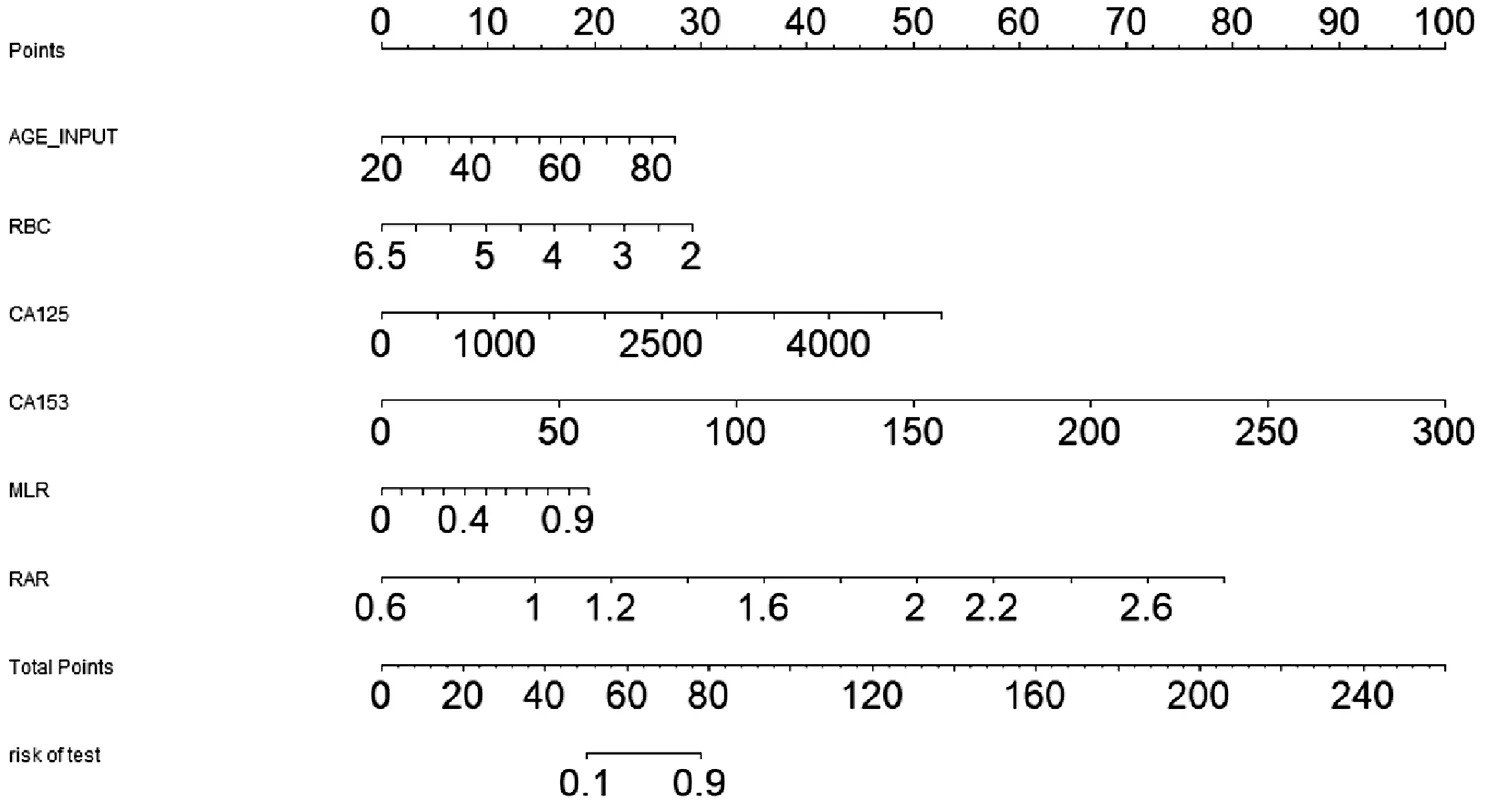
Nomogram of differential diagnosis. Calculate the sub-scores for each of the six indicators according to their respective point values, then sum these sub-scores to get the total score. This total score represents the individual’s comprehensive evaluation under the model

Ultimately, six core predictors-age, RBC count, CA125, CA153, RAR, and MLR, were retained for model construction. A nomogram was developed based on them, illustrated in Fig. 2.

**Fig. 3:**
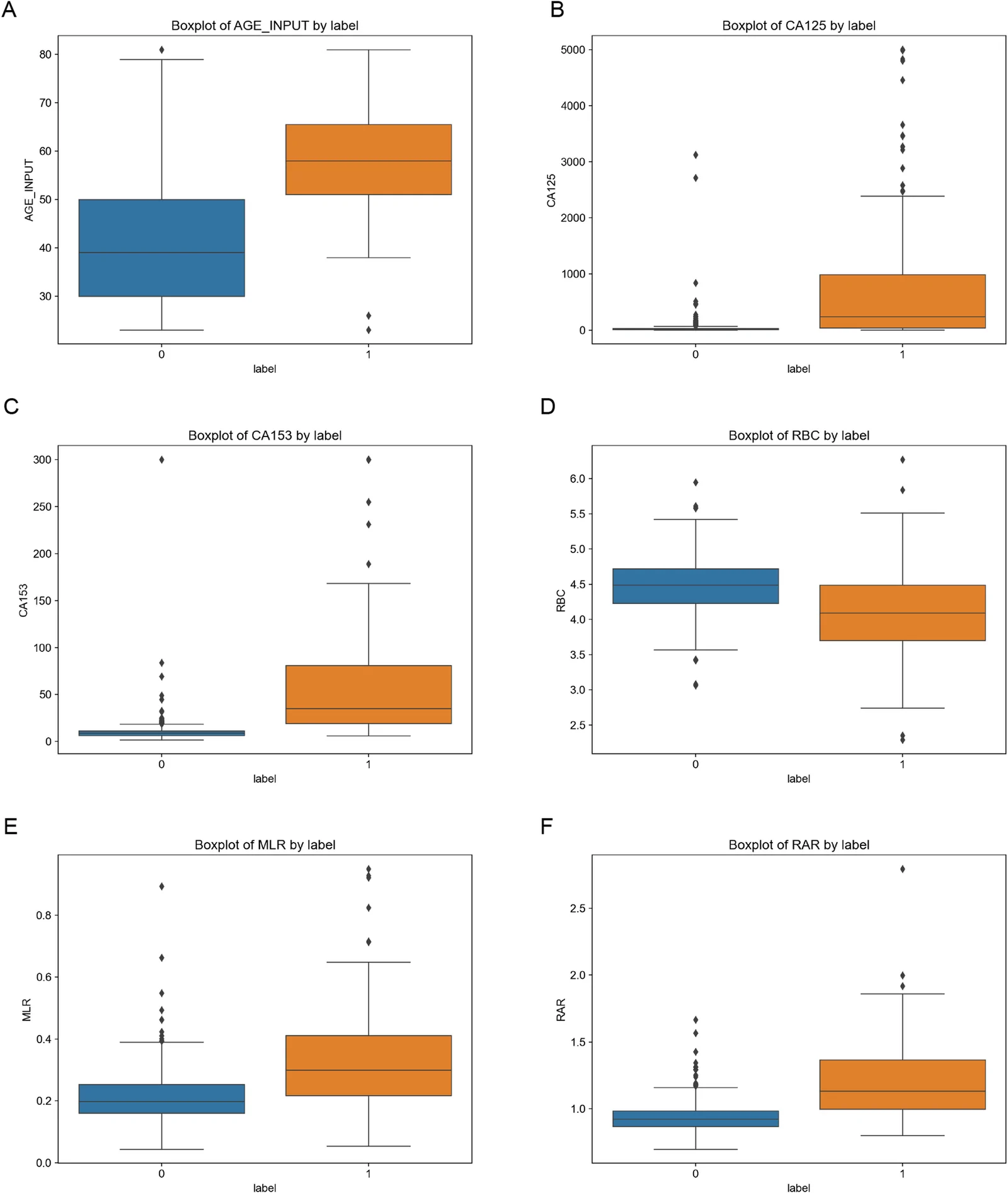
Box plots of the six core variables comparing benign and malignant groups. Respectively, (**A**) Age. (**B**) CA125. (**C**) CA153. (**D**) RBC. (**E**) MLR, and (**F**) RAR. Significant differences were observed between the two groups for all six variables

Finally, a total of 648 patients were included, and their baseline characteristics were summarized in supplemental Table 5. The median age of patients in the SOC group (*n* = 155) was significantly higher than that of patients in the benign cyst group (*n* = 493) (57 years vs. 39 years, *P* < 0.001). As expected, tumor markers CA125 and CA153 were significantly elevated in the SOC group (both *P* < 0.001). Regarding inflammatory, immune, and derived indicators, SOC patients exhibited a pronounced systemic inflammatory response and compromised immune-nutritional status, as evidenced by significantly higher MLR and RAR levels and significantly lower RBC counts (all *P* < 0.001). The results of the intergroup comparisons for the six core predictive variables were presented in Fig. 3(A-F).

### Feature selection and model performance comparison

**Fig. 4:**
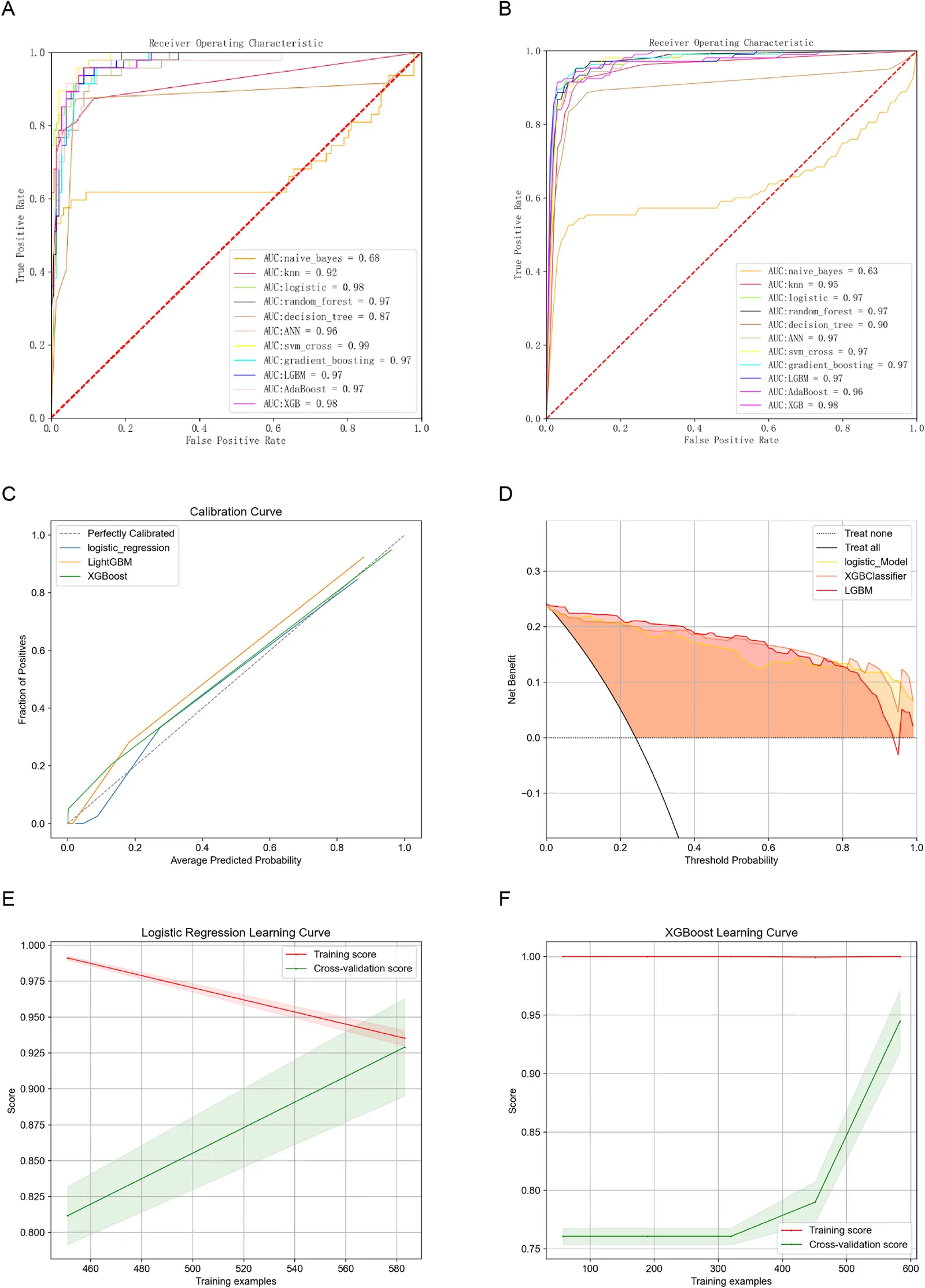
Model screening path diagram. (**A**) ROC curves of 11 machine learning models in the validation cohort. (**B**) ROC curves of the 11 models in the cross-validation set. Based on the AUC values from these two evaluation methods, three models- LR, LGBM, and XGBoost- were selected from the validation cohort for further assessment. Calibration curve (**C**) and the DCA curve (**D**) of the LR, LGBM and XGBoost models. The LGBM model demonstrated the lowest net benefit and was therefore excluded. The performance of the LR algorithm model and the XGBoost algorithm model on the learning curve are as shown in Fig. 4E and F respectively. In terms of performance on the learning curve, the LR model has a better fit and performs the best

In the internal validation cohort (*n* = 195), ROC curve analysis and cross-validation were performed to identify the top performing models from 11 machine learning algorithms (Fig. 4A and B). These candidate models were subsequently evaluated using calibration curves and DCA (Fig. 4C and D). The AdaBoost model exhibited the lowest clinical utility and was consequently excluded. The LR and XGBoost models were further assessed using precision-recall curves, calibration curves, DCA, and learning curves (Fig. 4E and F). The discriminative performance of all 11 machine learning models regarding discriminative ability (including optimal threshold, accuracy, positive precision, negative precision, positive recall, negative recall, F1 score, kappa, AUC, AUC 95% CI) was summarized in supplemental Table 6. Ultimately, LR was selected as the final model because it achieved performance comparable to the best-performing algorithms while offering superior interpretability and clinical utility. The final LR model demonstrated an AUC of 0.979 (95% CI: 0.958–0.994), sensitivity of 89.4%, specificity of 93.9%, accuracy of 92.8%, F1 score of 92.9%, and Kappa coefficient of 80.9%. The XGBoost and LightGBM ranked next in performance, whereas the Naive Bayes (NB) model showed the lowest discriminative ability (AUC = 0.677), highlighting the superiority of more complex machine learning algorithms in capturing nonlinear relationships among variables.

### Calibration and clinical utility of the optimal model

**Fig. 5:**
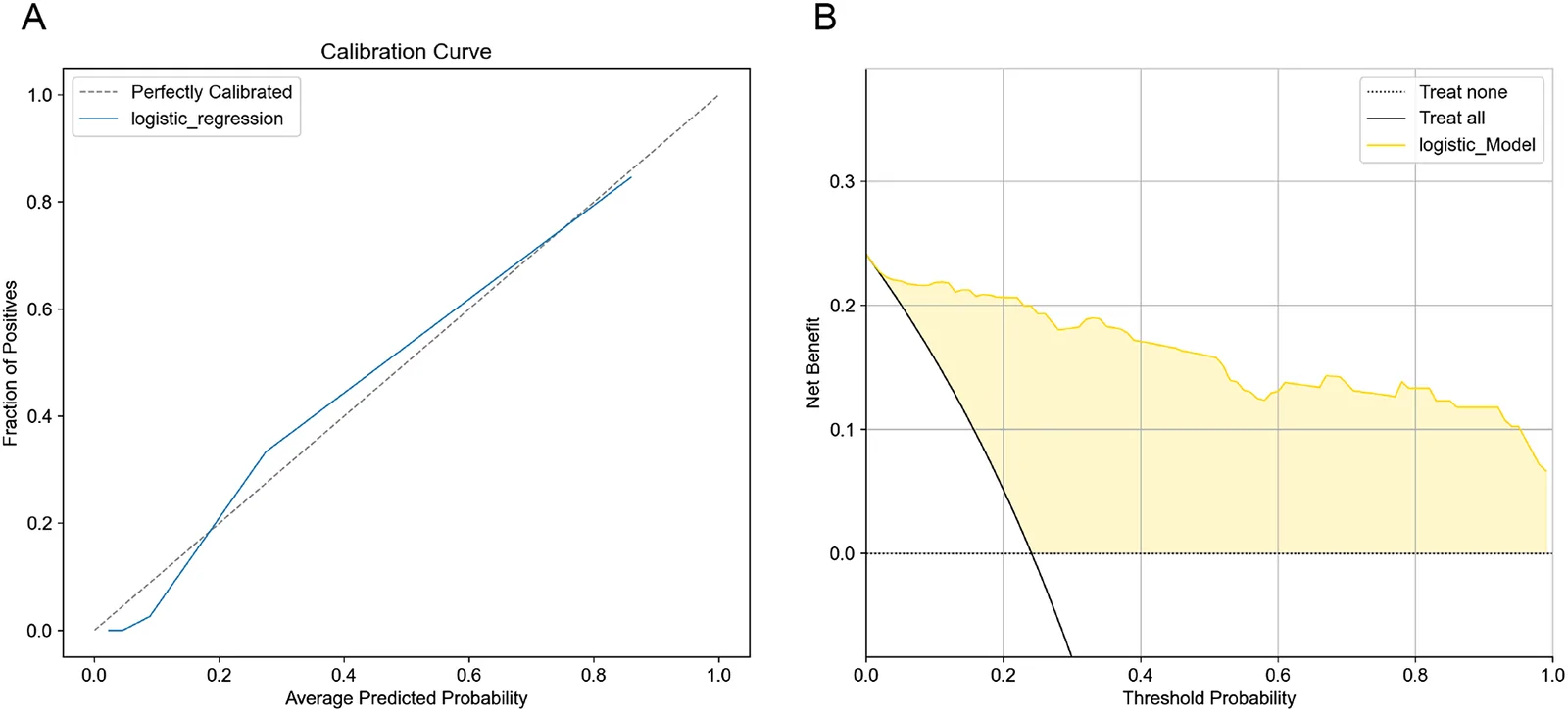
Calibration curve and DCA curve of the LR model. (**A**) Calibration curve showing that the predicted probabilities are highly consistent with the observed actual risk, with a Brier score of 0.065, indicating good model calibration, (**B**) The DCA curve demonstrating that the LR model yields greater net clinical benefit than the strategies of “treat all as malignant” or “treat all as benign” across a range of threshold probabilities

The optimal LR model not only demonstrated excellent discriminative performance but also exhibited strong calibration, as shown by the calibration curve Fig. 5(A), with a Brier score of 0.065, indicating high consistency between predicted probabilities and observed outcomes. As shown in Fig. 5B, DCA showed that the LR model delivered greater net clinical benefit than both the treat-all and treat-none strategies across threshold probabilities of 0.1–0.8 .

### Model interpretation: SHAP analysis

**Fig. 6:**
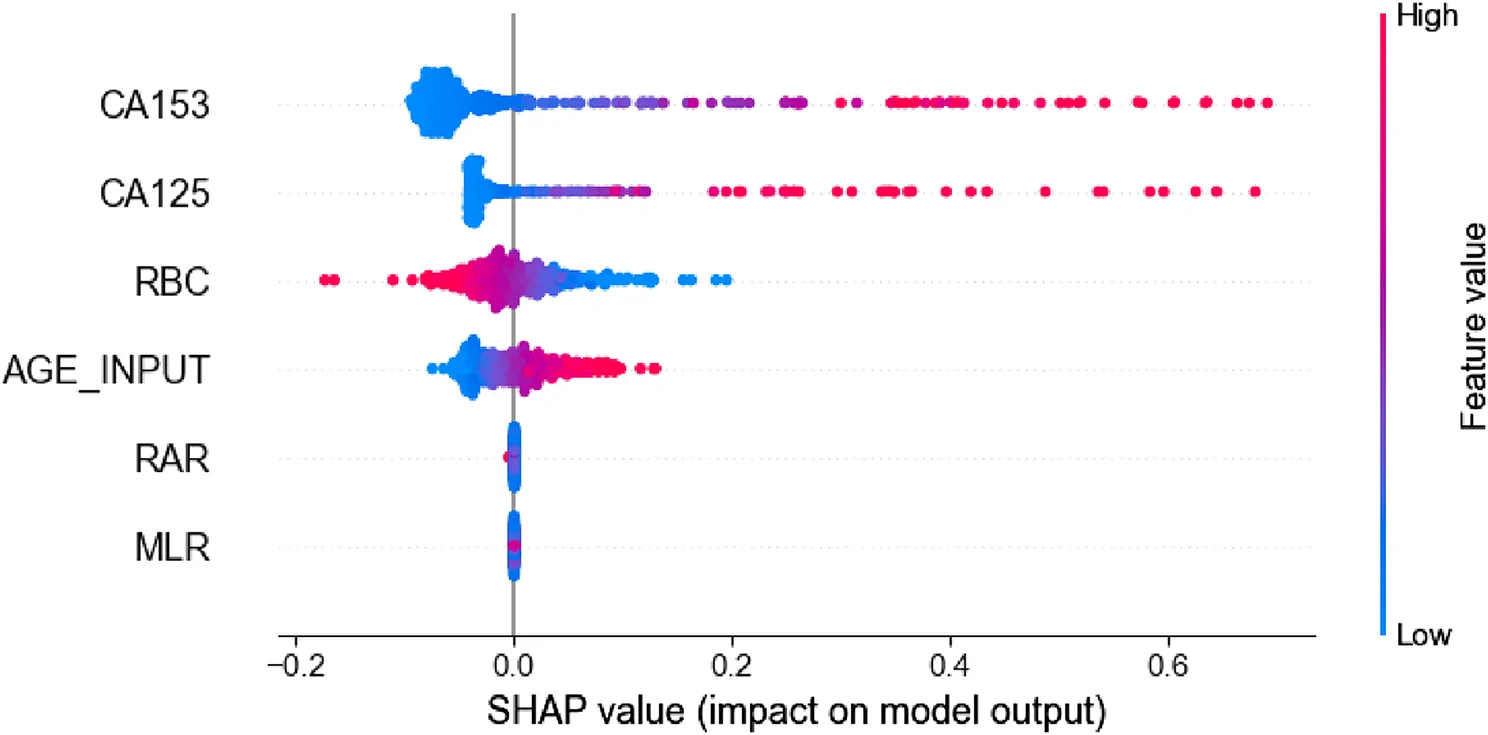
A globally interpretable SHAP summary plot. CA153 is the most influential predictor, elevated levels (red dot) are strongly associated with positive SHAP value, driving the prediction toward the “malignant” direction. CA125 and RBC followed closely. Higher age, CA125, CA153, RAR, and MLR are the primary drivers of accurate malignant predictions. Conversely, RBC serves as a protective factor, with higher levels (red points) shifting predictions toward benign classification

The SHAP summary plot (Fig. 6) presents a global interpretation of all predictors, ranked by their mean absolute impact on the model output. CA153 was identified as the most influential predictor; its elevated levels (red points) consistently yielded positive SHAP values, thereby exerting the strongest propensity toward malignant classification. CA125 and RBC ranked second and third in importance, respectively. Notably, RBC served as a key protective factor, wherein lower levels (blue points) were associated with positive SHAP values and thus contributed to malignancy prediction. Overall, advanced age, together with elevated CA125, CA153, MLR, and RAR levels constituted the primary drivers of malignant classification, while high RBC levels were the primary basis for predicting benign outcomes.

## Discussion

This study, based on single-center retrospective data, developed and validated an interpretable machine learning model integrating six clinical routine indicators (age, CA125, CA153, RBC, MLR, RAR) for differentiating serous ovarian cancer and benign ovarian cysts. The LR model demonstrated excellent discriminatory performance (AUC: 0.979) and good calibration, with predictions transparently explained through SHAP analysis. Our primary contributions are threefold: expanding the feature space for SOC differential diagnosis beyond traditional biomarkers, demonstrating that a simple linear model can achieve high accuracy with carefully selected routine indicators, and providing individualized interpretability through SHAP to facilitate clinical adoption.

Compared with the ROMA index (which relies on CA125, HE4, and menopausal status), our model offers several potential advantages: First, it eliminates the requirement for HE4 testing, which is not universally available, particularly in resource-limited regions; Second, by integrating inflammatory and nutritional markers (MLR and RAR), our model may improve specificity in complex benign conditions such as endometriosis, pelvic inflammatory disease, where CA125 and HE4 can be falsely elevated. Third, unlike ROMA’s fixed algorithm, our SHAP-based approach provides individualized explanations for each prediction, enabling clinicians to understand which specific factors drive the malignancy risk for individual patients. We acknowledge that a direct head-to-head comparison with ROMA was precluded by the high missing rate of HE4 data (> 20%) in our retrospective database. Future prospective studies should include formal non-inferiority or superiority comparisons.

We significantly expanded the feature dimensions for SOC differential diagnosis, moving from traditional 2–3 markers to a comprehensive set of 54 clinical indicators spanning inflammatory response, coagulation function, hepatic and renal function, and metabolic status. Among these, inflammatory and immune-derived markers (MLR and RAR) demonstrated notable diagnostic value. Both markers were significantly elevated in SOC patients, reflecting the critical role of systemic immune-inflammatory responses in cancer progression [24]. Studies have demonstrated that circulating monocytes promote tumor angiogenesis, invasion, and metastasis through their differentiation into tumor-associated macrophages (TAMs), whereas a decline in lymphocytes—especially cytotoxic T cells—signals compromised anti-tumor immunity [25]. MLR comprehensively reflects the dynamic interplay between pro-tumor and anti-tumor immune mechanisms [26], and meta-analyses have confirmed its prognostic value across solid tumors [27]. Similarly, RAR—combining RDWSD and ALB—reflects nutritional-metabolic and inflammatory status. While previous studies have examined RAR components within metabolic-nutritional-inflammation scores [28]. Our study innovatively identified that the combination of this composite marker, RAR, holds unique diagnostic value in distinguishing benign from malignant ovarian diseases, potentially due to its ability to capture abnormalities across both inflammatory and nutritional-metabolic dimensions. Recent studies have also confirmed the prognostic significance of RAR in diabetic foot ulcers [29]. A prospective study published in 2024 further demonstrated that a scoring system incorporating inflammatory and nutritional indicators can effectively predict chemotherapy response and survival outcomes in ovarian cancer patients [30]. Consequently, multidimensional data analysis allows for a more comprehensive characterization of disease-related physiological and pathological changes, thereby enhancing diagnostic accuracy.

Another key finding was the protective role of RBC count. Traditional tumor-marker studies have predominantly focused on leukocyte or platelet indices. Interestingly, our SHAP analysis revealed that higher RBC counts were associated with reduced malignancy likelihood. This likely reflects cancer-related anemia (anemia of chronic disease, ACD), which arises when SOC disrupts iron metabolism through various inflammatory cytokines (e.g., IL-6, hepcidin), inhibits erythropoiesis, and shortens erythrocyte lifespan [31]. Additionally, tumor-related malnutrition and chronic blood loss may further contribute to anemia [32]. Thus, RBC should be viewed not merely as a routine blood parameter but as a comprehensive indicator of tumor burden and physiological dysregulation [33]. This interpretation aligns with evidence that perioperative blood transfusions may negatively impact ovarian cancer survival, supporting restrictive transfusion strategies during cytoreductive surgery and adhering to evidence-based transfusion guidelines to reduce transfusion rates in this patient population, thereby improving clinical outcomes [32]. The biological mechanisms linking these parameters to tumor development warrant further investigation. In particular, cancer-related metabolic reprogramming and systemic metabolic alterations may contribute to these associations. Previous studies have shown that dysregulation of lipid metabolism and metabolic homeostasis contributes to tumor progression and tumor–host interactions. As highlighted by Modica et al., dysregulation of systemic metabolic homeostasis, including alterations in iron metabolism and inflammatory-mediated nutritional depletion, plays a crucial role in tumor progression and tumor-host interactions [34].

For model development, we employed an integrated feature selection strategy combining LASSO regression with tree-based feature importance assessment. This approach effectively mitigated overfitting risk and identified the most clinically relevant core indicators. Unlike previous studies relying on a single feature selection method, our strategy significantly enhanced both robustness and interpretability [35]. Notably, all six selected features were derived from routine clinical tests, requiring no additional costs or specialized equipment. This ensures broad applicability across medical institutions at all levels, representing a clear advantage over approaches that depend on costly genomic profiling or complex imaging features [36].

Although we did not empirically train deep learning (DL) models in this study (as our dataset size and feature dimensionality were better suited for traditional ML), we contextualize our findings within the DL literature. DL frameworks (e.g., 1D-Convolutional Neural Network (CNN), transformers) excel at extracting high-dimensional features from imaging or omics data but typically require large sample sizes and substantial computational resources [37]. In contrast, our LR model achieved comparable performance (AUC: 0.979) using only 6 routine clinical indicators, avoiding DL’s dependency on specialized data (e.g., genomic profiles) or heavy computing infrastructure. This comparison is based on published external metrics [13, 14, 36], rather than direct head-to-head evaluation. Interestingly, the XGBoost model also achieved excellent performance (AUC: 0.979) in our cohort, suggesting that within the low-dimensional feature space formed by these six strong predictors, even simple linear models can attain high accuracy. This has significant practical implications: AI tools based on simpler models can perform effectively in clinical settings with limited computational resources, thereby lowering the deployment barrier in resource-constrained environments [38, 39].

A distinctive strength of this study is the use of SHAP for model interpretability. Traditional machine learning models are often criticized as “black boxes,” which limits their adoption and trust by clinicians [40]. SHAP values quantitatively measure each feature’s contribution to individual predictions while clarifying the effect. For example, the SHAP summary plots revealed CA153 as the most influential predictor elevating malignancy risk, whereas RBC served as a protective factor. This transparency enables clinicians to evaluate whether the model’s reasoning aligns with medical knowledge, fostering greater confidence in clinical adoption [41]. Moreover, SHAP dependence plots can reveal potential nonlinear relationships that traditional LR models cannot capture [42].

Several limitations must be acknowledged. First, our study was retrospective and single-institutional, although we used random 7:3 splitting and 5-fold cross-validation for hyperparameter tuning, performance estimates derived from a single hold-out set remain vulnerable to sampling variability and overfitting given the ratio of candidate predictors (*n* = 54) to the event rate (*n* = 155 SOC cases). External validation on independent, multi-center cohorts with diverse demographics, laboratory protocols, and disease spectra is essential. Second, the retrospective design introduces several biases: (i) selection bias, as our tertiary hospital setting likely introduces referral bias, enriching for complex or severe cases that may not represent community populations; (ii) spectrum bias: due to the lack of temporal validation to assess shifts in patient characteristics over time; (iii) residual confounding from unmeasured variables (e.g., genetic mutations, exact tumor stage, socioeconomic status); and (iv) verification bias, minimized but not eliminated by dual pathological review as the gold standard, with potential inter-observer variability across institutions. External validation with completely independent multi-center data is necessary and constitutes the next critical direction of our future work. Third, high missing rates for some predictors (e.g., HE4) precluded their inclusion. Fourth, our model focused specifically on differentiating SOC from benign cysts, excluding other ovarian malignancies (e.g., mucinous carcinoma, endometrioid carcinoma) and borderline tumors, its performance in these populations remains unknown. Finally, although the model demonstrated excellent discrimination, its real-world impact on clinical outcomes—such as surgical decision-making or survival—requires further evaluation through implementation studies.

Based on these limitations, we propose several directions for future research: First, prospective multi-center studies should be conducted to validate the generalizability and explore integration with imaging features to develop a multimodal diagnostic framework [43]. Second, the model should be incorporated into a clinical decision support system (CDSS), with cluster randomized controlled trials assessing its real-world impact on clinical decision-making quality, patient outcomes, and healthcare costs [44]. Third, the relationship between hematological indicators and tumor molecular features (e.g., homologous recombination deficiency status and BRCA mutation profiles) warrants investigation to yield novel insights into the pathogenesis of ovarian cancer [45]. Finally, a simplified, mobile-compatible version should be developed and evaluated as a primary screening tool in primary care and resource-limited settings.

## Conclusion

In conclusion, this study proposes a high-precision, interpretable machine learning model for differentiating serous ovarian cancer from benign ovarian cysts using routine laboratory tests. The model shows good performance, suggesting that it could be effectively used for preoperative differential diagnosis, thereby assisting clinicians in optimizing patient management and reducing unnecessary interventions. An open web tool based on six core variables was developed to support clinical diagnosis. The tool is available at: https://sdsl-soc-hkxguyxoejsi4hhrkqrzfq.streamlit.app/.

## Supplementary Information

Below is the link to the electronic supplementary material.

Supplementary Material 1

Supplementary Material 2

Supplementary Material 3

Supplementary Material 4

Supplementary Material 5

Supplementary Material 6

## Data Availability

All data generated or analyzed during this study are included in this article and supplementary information files. The commercial i-Research software, developed by Roche based on Python and R languages was used in this study for the statistical analysis. The model files and the source code of the web page have been uploaded to the GitHub repository. The address is as follows: https://github.com/weicheng1104/precise-discrimination-between-serous-ovarian-cancer-and-ovarian-cysts.

## Abbreviations

WBC: White blood cell count
RBC: Red blood cell count
HGB: Hemoglobin
PLT: Platelet count
NEUT#: Absolute neutrophil count
LYMPH#: Absolute lymphocyte count
MONO#: Absolute monocyte count
EO#: Absolute eosinophil count
BASO#: Absolute basophil count
NEUT%: Percentage of neutrophils
LYMPH%: Percentage of lymphocytes
MONO%: Percentage of monocytes
EO%: Percentage of eosinophil
BASO%: Percentage of basophil
HCT: Hematocrit
MCV: Mean corpuscular volume
MCH: Mean corpuscular hemoglobin
MCHC: Mean corpuscular hemoglobin concentration
RDWCV: Red blood cell distribution width coefficient of variation
RDWSD: Red blood cell distribution width standard deviation
TP: Total protein
ALB: Albumin
GLOB: Globulin
A/G: Albumin/globulin ratio
TBIL: Total bilirubin
DBIL: Direct bilirubin
IBIL: Indirect bilirubin
ALT: Alanine aminotransferase
AST: Aspartate aminotransferase
GGT: Gamma-glutamyl transferase
ALP: Alkaline phosphatase
LDH: Lactate dehydrogenase
GLDH: Glutamate dehydrogenase
ADA: Adenosine deaminase
TBA: Total bile acid
BUN: Blood urea nitrogen
CREA: Creatinine
BUN/CREA: BUN/creatinine ratio
UA: Uric acid
Cys-C: Cystatin C
AFP: Alpha fetoprotein
CEA: Carcinoembryonic antigen
CA125: Carbohydrate antigen 125
CA153: Carbohydrate antigen 153
CA724: Carbohydrate antigen 724
NLR: Neutrophil-to-lymphocyte ratio
PLR: Platelet-to-lymphocyte ratio
MLR: Monocyte-to-lymphocyte ratio
SII: Systemic immune-inflammation index
NPAR: Neutrophil percentage-to-albumin ratio
RAR: Red blood cell distribution width standard deviation-to-albumin ratio
HALP: Hemoglobin-albumin-lymphocyte-platelet score
SIRI: Systemic inflammation response index
AISI: Aggregate index of systemic inflammation
