# Supplementary material for: An interpretable machine learning model integrating 54 test indicators and age: a single-center study on precise discrimination between serous ovarian cancer and ovarian cysts
Source: BMC Med Inform Decis Mak. 2026 Aug 8;26:453. doi: 10.1186/s12911-026-03727-3 (PMC13741575; doi:10.1186/s12911-026-03727-3)
Supplement: Supplementary file 1 [file 12911_2026_3727_MOESM1_ESM.docx]

**Supplemental table 1: Flowchart of Research Ideas**

|  | Patients with serous ovarian cancer (SOC) and ovarian cysts in Shandong Provincial Hospital Affiliated to Shandong First Medical University from 2016 to 2022 were collected. | | | | |  |  |
| --- | --- | --- | --- | --- | --- | --- | --- |
|  |  |  |  |  |  |  |  |
|  |  |  |  |  |  |  |  |
|  |  |  |  |  |  | Inclusion and exclusion. |  |
|  |  |  |  |  |  |  |  |
|  | A total of 648 patients, including 155 patients with SOC and 493 patients with ovarian cysts, were included in the cohort. | | | | |  | 1,Data collection preparation. |
|  |  |  |  |  |  |  |  |
|  |  |  |  |  |  |  |  |
|  |  |  |  |  |  |  |  |
| The training cohort accounts for 70%, which is calculated as 453 cases. | | |  | The internal validation queue accounts for 30%, which is calculated as 195 cases. | | |  |
|  |  |  |  |  |  |  |  |
|  |  |  |  |  |  |  |  |
|  |  |  |  |  |  |  |  |
|  |  |  |  |  |  |  |  |
|  | A total of 54 items were collected, including the patient's age, 45 test indicators such as blood routine, biochemistry, and coagulation indicators, as well as 9 derivative indicators based on these test indicators. | | | | |  |  |
|  |  |  |  |  |  |  |  |
|  |  |  |  |  |  |  |  |
|  |  |  |  |  |  |  |  |
|  |  |  |  |  |  |  |  |
|  |  |  |  |  |  |  |  |
|  | Blank values are filled with median values. | | | | |  |  |
|  |  |  |  |  |  |  |  |
|  |  |  |  |  |  |  |  |
|  | Feature selection: Seven indicators were screened using LASSO regression, and then the white ball ratio indicator was excluded by using the feature importance of random forest and decision tree. | | | | |  | 2.Model development and internal validation |
|  |  |  |  |  |  |  |  |
|  |  |  |  |  |  |  |  |
|  |  |  |  |  |  |  |  |
|  |  |  |  |  |  |  |  |
|  | Model development. | |  | Internal verification. | |  |  |
|  |  |  |  |  |  |  |  |
|  | The logic was evaluated through eight evaluation tools such as the ROC curve and calibration curve Eleven algorithms such as regression, random forest, and XGBoost were evaluated.Find the optimal model from it. | | | | |  |  |
|  |  |  |  |  |  |  |  |
|  |  |  |  |  |  |  |  |
|  |  |  |  |  |  |  |  |
|  |  |  |  |  |  |  | 3. Model Interpretation and Application. |
|  |  |  |  |  |  |  |  |
|  | Explanation of the SHAP model and its application based on clinical development. | | | | |  |  |
|  |  |  |  |  |  |  |  |
|  |  | | | | |  |  |
