# Supplementary material for: An interpretable machine learning model integrating 54 test indicators and age: a single-center study on precise discrimination between serous ovarian cancer and ovarian cysts
Source: BMC Med Inform Decis Mak. 2026 Aug 8;26:453. doi: 10.1186/s12911-026-03727-3 (PMC13741575; doi:10.1186/s12911-026-03727-3)
Supplement: Supplementary file 2 [file 12911_2026_3727_MOESM2_ESM.docx]

**Supplemental table 2. Twenty four items with missing rate<10%**

| indicators | missing values |
| --- | --- |
| **CEA** | 3.24% |
| **CA153** | 2.16% |
| **CA125** | 1.23% |
| **ADA** | 1.08% |
| **CYSC** | 0.93% |
| **RDW/ALB** | 0.77% |
| **BUN/CREA** | 0.62% |
| **TP** | 0.62% |
| **URIC** | 0.62% |
| **ALT** | 0.62% |
| **ALP** | 0.62% |
| **GLDH** | 0.62% |
| **CREA** | 0.62% |
| **TBA** | 0.62% |
| **ALB** | 0.62% |
| **BUN** | 0.62% |
| **GLO** | 0.62% |
| **A/G** | 0.62% |
| **NPAR** | 0.62% |
| **DBIL** | 0.62% |
| **IBIL** | 0.62% |
| **TBIL** | 0.62% |
| **AST** | 0.62% |
| **GGT** | 0.62% |
