# Supplementary material for: An interpretable machine learning model integrating 54 test indicators and age: a single-center study on precise discrimination between serous ovarian cancer and ovarian cysts
Source: BMC Med Inform Decis Mak. 2026 Aug 8;26:453. doi: 10.1186/s12911-026-03727-3 (PMC13741575; doi:10.1186/s12911-026-03727-3)
Supplement: Supplementary file 3 [file 12911_2026_3727_MOESM3_ESM.docx]

**Supplemental table 3. Single-factor and Multi-factor Analysis**

| **Variable** | **Missing_Count** | **Total_Count** | **Label_BG_Count** | **Label_MG_Count** | **P_Value_Single_Factor** | **P_Value_Multi_Factor** |
| --- | --- | --- | --- | --- | --- | --- |
| AGE_INPUT | 0 | 648 | 493 | 155 | 1.85E-46 | 0.000193 |
| RBC | 0 | 648 | 493 | 155 | 6.05E-11 | 2.28E-07 |
| CA125 | 0 | 648 | 493 | 155 | 1.92E-12 | 0.003534 |
| CA153 | 0 | 648 | 493 | 155 | 6.28E-16 | 0.005576 |
| MLR | 0 | 648 | 493 | 155 | 3.48E-16 | 1.11E-05 |
| RAR | 0 | 648 | 493 | 155 | 7.47E-22 | 0.051059 |
