# Supplementary material for: An interpretable machine learning model integrating 54 test indicators and age: a single-center study on precise discrimination between serous ovarian cancer and ovarian cysts
Source: BMC Med Inform Decis Mak. 2026 Aug 8;26:453. doi: 10.1186/s12911-026-03727-3 (PMC13741575; doi:10.1186/s12911-026-03727-3)
Supplement: Supplementary file 4 [file 12911_2026_3727_MOESM4_ESM.docx]

**Supplemental table 4. Independent T-Test**

| **Variable** | **BG(mean)** | **BG(SD)** | **MG(mean)** | **MG(SD)** | **T_Value** | **P_Value** |
| --- | --- | --- | --- | --- | --- | --- |
| AGE_INPUT | 41.12779 | 12.94084 | 58.31613 | 10.3213 | -16.9611 | 1.85E-46 |
| RBC | 4.47858 | 0.375895 | 4.111613 | 0.623851 | 6.938112 | 6.05E-11 |
| CA125 | 46.99381 | 194.4521 | 943.1749 | 1401.971 | -7.93438 | 3.86E-13 |
| CA153 | 10.68529 | 14.61187 | 70.98277 | 78.32497 | -9.53239 | 2.76E-17 |
| MLR | 0.214314 | 0.080532 | 0.335084 | 0.16103 | -8.99043 | 3.48E-16 |
| RAR | 0.937489 | 0.109031 | 1.209703 | 0.286741 | -11.5594 | 4.05E-23 |
