# Supplementary material for: An interpretable machine learning model integrating 54 test indicators and age: a single-center study on precise discrimination between serous ovarian cancer and ovarian cysts
Source: BMC Med Inform Decis Mak. 2026 Aug 8;26:453. doi: 10.1186/s12911-026-03727-3 (PMC13741575; doi:10.1186/s12911-026-03727-3)
Supplement: Supplementary file 5 [file 12911_2026_3727_MOESM5_ESM.docx]

**Supplemental table 5. Comparison of baseline Characteristics between Patients with serous ovarian cancer (SOC) and those with benign ovarian cysts [M(P_25_, P_75_)]**

| **Characteristic** | **Overall (N=648)** | **SOC (n=155)** | **Benign Cyst (n=493)** | ***P* value** |
| --- | --- | --- | --- | --- |
| Age, years | 45(32, 55) | 57(51, 64) | 39(30, 50) | <0.001 |
| Tumor Markers |  |  |  |  |
| CA125, U/mL | 22.8(12.7, 72.2) | 262.2(41.9, 1013.5) | 17.9(11.2, 34.9) | <0.001 |
| CA153, U/mL | 10.3(7.3, 17.6) | 38.6(21.2, 88.2) | 8.9(6.6, 11.5) | <0.001 |
| Inflammation & Immune |  |  |  |  |
| RBC, ×10^12^ | 4.4(4.1,4.7) | 4.1(3.7, 4.5) | 4.5(4.0, 4.7) | <0.001 |
| MLR | 0.22(0.17, 0.28) | 0.30(0.21, 0.41) | 0.20(0.16, 0.25) | <0.001 |
| RAR | 0.95(0.88, 1.05) | 1.14(1.00, 1.37) | 0.92(0.87, 0.99) | <0.001 |
