# Supplementary material for: An interpretable machine learning model integrating 54 test indicators and age: a single-center study on precise discrimination between serous ovarian cancer and ovarian cysts
Source: BMC Med Inform Decis Mak. 2026 Aug 8;26:453. doi: 10.1186/s12911-026-03727-3 (PMC13741575; doi:10.1186/s12911-026-03727-3)
Supplement: Supplementary file 6 [file 12911_2026_3727_MOESM6_ESM.docx]

**Supplemental table 6. Performance Comparison of various machine learning models on the internal validation set**

| **name** | **optimal_threshold** | **Accuracy** | **Positive Precision** | **Negative Precision** | **Positive Recall** | **Negative Recall** | **F1_score** | **kappa** | **AUC** | **AUC 95% CI** |
| --- | --- | --- | --- | --- | --- | --- | --- | --- | --- | --- |
| logistic_regression | 0.240 | 0.928 | 0.823529412 | 0.965277778 | 0.893617021 | 0.939189189 | 0.9291786 | 0.8093043 | 0.97886717 | 0.9582881171116465-0.9940342925481448 |
| XGBoost | 0.048539191 | 0.918 | 0.771929825 | 0.97826087 | 0.936170213 | 0.912162162 | 0.920459 | 0.7909127 | 0.9787234 | 0.9574792517006803-0.9934372514536258 |
| LightGBM | 0.074073508 | 0.913 | 0.767857143 | 0.971223022 | 0.914893617 | 0.912162162 | 0.9152624 | 0.7763309 | 0.97441058 | 0.9557488517313091-0.9897563066139171 |
| random_forest | 0.22 | 0.913 | 0.75862069 | 0.97810219 | 0.936170213 | 0.905405405 | 0.9157046 | 0.7793383 | 0.97347614 | 0.9472566440805208-0.9927795309188099 |
| AdaBoost | 0.486867797 | 0.928 | 0.8 | 0.978571429 | 0.936170213 | 0.925675676 | 0.9300235 | 0.814538 | 0.97096032 | 0.9393564041623843-0.9936353901652177 |
| GradientBoost | 0.067074032 | 0.923 | 0.807692308 | 0.965034965 | 0.893617021 | 0.932432432 | 0.9243586 | 0.7971145 | 0.96866015 | 0.9461604781501866-0.9874734477124183 |
| ANN | 0.145268934 | 0.908 | 0.754385965 | 0.971014493 | 0.914893617 | 0.905405405 | 0.9105164 | 0.7647768 | 0.9626222 | 0.9331371577780523-0.9838509087685738 |
| SVM | 0.199618405 | 0.897 | 0.764705882 | 0.944444444 | 0.829787234 | 0.918918919 | 0.8988265 | 0.7275775 | 0.95457159 | 0.9171824221406228-0.9805695261993593 |
| KNN | 0.2 | 0.897 | 0.764705882 | 0.944444444 | 0.829787234 | 0.918918919 | 0.8988265 | 0.7275775 | 0.91697815 | 0.8545715438073962-0.9690171103631171 |
| decision_tree | 0.625 | 0.917948718 | 0.816326531 | 0.952054795 | 0.85106383 | 0.939189189 | 0.9185243 | 0.7789429 | 0.86975273 | 0.7792948917092666-0.9455646175898061 |
| naive_bayes | 3.15E-11 | 0.856410256 | 0.771428571 | 0.875 | 0.574468085 | 0.945945946 | 0.8487009 | 0.5700787 | 0.67653824 | 0.5529972715318869-0.790988612082362 |
